# Expression profiling in APP23 mouse brain: inhibition of Aβ amyloidosis and inflammation in response to LXR agonist treatment

**DOI:** 10.1186/1750-1326-2-20

**Published:** 2007-10-22

**Authors:** Iliya Lefterov, Angie Bookout, Zhu Wang, Matthias Staufenbiel, David Mangelsdorf, Radosveta Koldamova

**Affiliations:** 1 Department of Environmental and Occupational Health, Graduate School of Public Health, University of Pittsburgh, Pittsburgh, PA 15219, USA; 2 Department of Pharmacology, Howard Hughes Medical Institute, University of Texas Southwestern Medical Center, Dallas, TX 75390, USA; 3 Department of Nervous System, Novartis Institutes of BioMedical Research, CH-4002 Basel, Switzerland

## Abstract

**Background:**

Recent studies demonstrate that in addition to its modulatory effect on APP processing, *in vivo *application of Liver X Receptor agonist T0901317 (T0) to APP transgenic and non-transgenic mice decreases the level of Aβ_42. _Moreover, in young Tg2576 mice T0 completely reversed contextual memory deficits. Compared to other tissues, the regulatory functions of LXRs in brain remain largely unexplored and our knowledge so far is limited to the cholesterol transporters and apoE. In this study we applied T0 to APP23 mice for various times and examined gene and protein expression. We also performed a series of experiments with primary brain cells derived from wild type and LXR knockout mice subjected to various LXR agonist treatments and inflammatory stimuli.

**Results:**

We demonstrate an upregulation of genes related to lipid metabolism/transport, metabolism of xenobiotics and detoxification. Downregulated genes are involved in immune response and inflammation, cell death and apoptosis. Additional treatment experiments demonstrated an increase of soluble apolipoproteins E and A-I and a decrease of insoluble Aβ. In primary LXR^wt ^but not in LXRα^-/-^β^-/- ^microglia and astrocytes LXR agonists suppressed the inflammatory response induced by LPS or fibrillar Aβ.

**Conclusion:**

The results show that LXR agonists could alleviate AD pathology by acting on amyloid deposition and brain inflammation. An increased understanding of the LXR controlled regulation of Aβ aggregation and clearance systems will lead to the development of more specific and powerful agonists targeting LXR for the treatment of AD.

## Background

Recent studies have linked cholesterol metabolism and AD pathogenesis [1-3] but the molecular and physiological mechanisms remain elusive. The liver X receptors (LXRs), LXRα and LXRβ, are transcription factors that control the expression of genes involved in cholesterol metabolism and lipoprotein remodelling [4]. LXRα/β have been considered drug targets for cardiovascular disease and recent reports suggest that LXR ligands could also be therapeutic agents for inflammation and diabetes [5-9]. LXRα and LXRβ are activated by the same ligands but their tissue distribution differs: LXRα is highly expressed in liver, adipose tissue, and macrophages and moderately in the brain, while LXRβ is expressed in all tissues examined and is highly expressed in brain [10]. Compared to other tissues, the regulatory functions of LXRs in brain remain largely unexplored and our knowledge so far is limited to the cholesterol transporters and apoE. Remarkably *apoE*, a proven risk factor for sporadic AD, is an LXR target gene.

ATP-binding cassette transporter A1 (ABCA1), a major regulator of cholesterol efflux and generation of high density lipoproteins (HDL), is one of the most important LXR targets. It has been demonstrated that if ABCA1 is functionally impaired, poorly lipidated apoA-I in the periphery becomes unstable and is hyper-catabolized [11]. In brain, ABCA1 is considered essential for regulation of the internal cycling of cholesterol between glia and neurons. Recent data suggest that ABCA1 is essential also for apoE lipidation and for maintaining of its normal CNS concentration. Lack of ABCA1 in mice results in dramatically decreased CNS levels of apoE and abnormal structure of poorly lipidated lipoprotein complexes subjected to rapid degradation [12-16]. Recently we and two other groups have shown independently that ABCA1 deficiency increases Aβ deposition in different lines of APP transgenic mice accompanied by a substantial decrease in the level of brain apoE [12,14,16]. Keeping in mind the role of apoE in amyloid deposition, one explanation for this phenotype could be that insufficient and poorly lipidated apoA-I and apoE either decrease Aβ clearance or facilitate (apoE in particular) Aβ aggregation [12,14,16-18].

ABCA1 expression in neurons and glia, as well as its level in the whole brain is markedly increased after exposure to LXR ligands [19-23]. Furthermore, LXR ligands inhibit Aβ production *in vitro *[19-21,24,25] probably by affecting APP processing [20,24,25].

Studies on LXR activation in brain, have focused so far on APP models at young ages. It has been demonstrated that the administration of the synthetic LXR ligand T0 for one week to young pre-depositing APP, as well as wild type mice, increased the level of ABCA1 and decreased the levels of Aβ_40 _[21] and Aβ_42 _[19,21,26]. Moreover, the application of T0 for two weeks to Tg2576 completely reversed the contextual memory deficit in these mice which further supported the hypothesis that LXR agonists facilitate the clearance of Aβ [26]. Although it is not clear whether LXR activation can also prevent plaque formation, most recent data from the P. Tontonoz laboratory demonstrated that global deletion of LXRα or LXRβ in APP transgenic mice results in increased amyloid plaque load [27] which further strengthened the idea that LXR signalling and LXR responsive genes are important determinants of AD pathogenesis (at least deposition of fibrillar Aβ and its clearance) [14,21,27]. The present study was undertaken to reveal gene and protein expression in brain of 6-month old APP23 mice subjected to T0 treatment for various times. We also examined the levels of Aβ, and conducted experiments in isolated primary brain cells to further confirm the effects of LXR agonists on Aβ deposition and inflammatory reactions in mouse brain.

## Results

### Upregulation of ABCA1 and apolipoproteins in brain of T0 treated APP23 mice

**Figure 1:**
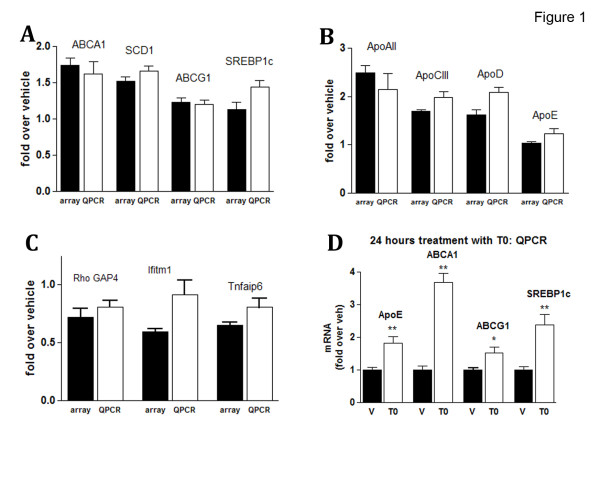
**Verification of array data**. **A, B and C**. 6-month old APP23 animals were treated with T0 for 25 days at a dose of 50 mg/kg/day (n = 5); control mice (n = 5) received vehicle and gene expression profiles were generated using Affymetrix 430A 2.0 mouse gene chips. RT-QPCR for selected up-regulated (**A and B**) and down-regulated (**C**) genes from T0-treated and control mice was performed on the same total RNA as used for the array assays and the results compared to those from the array experiments. Fold changes observed in arrays (white bars) and RT-QPCR (grey bars) for each gene tested are arranged adjacently (± S.E.M.) and were statistically significant (p < 0.05 using two-tailed Student's t test). **D**. 6–7 month old APP23 animals were treated for 24 hours with T0 (50 mg/kg body weight, n = 5); control mice (n = 5) received vehicle. Total RNA was extracted from homogenized cortices and hippocampi and the expression of selected LXR target genes was determined by RT-QPCR.

Previously we have demonstrated that short T0 treatment decreased the level of soluble Aβ in 3-month old pre-depositing APP23 mice in correlation with an increased ABCA1 level [21]. To determine the effect of activated LXRs on genes involved in the initial stages of Aβ deposition in older mice we treated 6-month old APP23 animals with T0 for 25 days (every day at a dose of 50 mg/kg body weight; control mice received vehicle; n = 5 for both groups). We specifically chose APP23 mice at an age when there is an increase in insoluble Aβ peptides, although actual amyloid plaques are still rare. At the end of the treatment total RNA was isolated from cortices and hippocampi and gene expression profiles for both groups were generated using Affymetrix 430A 2.0 mouse gene chips. The activation status of LXRs was confirmed by the increased level of ABCA1 mRNA of T0 versus vehicle treated mice as measured by RT-QPCR (Fig. 1A).

We identified 168 genes with fold change of at least 1.35 and p < 0.05 (*t*-test) which fall into the following main categories: Up-regulated – 75 genes coding for: (1) Lipid metabolism and transport; (2) Metabolism of xenobiotics and detoxification; (3) Stress response or response to stimulus; (4) Nuclear proteins and proteins involved in DNA binding or transcription. Down-regulated were 93 genes coding for: (1) Proteins involved in immune response and response to stress and inflammation; (2) Cell death and apoptosis; (3) Serine/threonine and tyrosine protein kinases; (4) Nuclear proteins known to participate in chromatin binding and remodelling, transcription and regulation of transcription. The first two clusters of up- or down-regulated genes are shown in Table 1. Results from RT-QPCR assays for selected up- and down-regulated genes are shown on Fig. 1A, B and 1C, and demonstrate that the fold change of all verified genes was statistically significant and validate the results.

**Table 1:** Differential expression of genes in brains of APP23 mice treated with T0 or vehicle. Fold change is expressed as ratio of vehicle versus T0 induced expression level, at p < 0.05.

**Fold change**	**Gene symbol**	**Gene product/cluster**
		**Up-regulated**
		**Lipid metabolism and transport**
		
4.60	Fabp1	fatty acid binding protein 1, liver
3.40	Acaa1b	acetyl-Coenzyme A acyltransferase 1B
2.70	Gc	group specific component (transport of vitamin D sterols)
2.27	Apoa2	apolipoprotein A-II
2.27	Apoc1	apolipoprotein C-I
1.75	Abca1	ATP-binding cassette, sub-family A (ABC1), member 1 (3 copies)
1.60	Hmgcs2	3-hydroxy-3-methylglutaryl-Coenzyme A synthase 2
1.59	Apod	apolipoprotein D
1.56	Cpt1a	carnitine palmitoyltransferase 1a, liver
1.49	Apoc3	apolipoprotein C-III
1.37	Scd1	stearoyl-Coenzyme A desaturase 1
		
		**Metabolism of xenobiotics and detoxification**
		
5.45	Cyp3a11	cytochrome P450, family 3, subfamily a, polypeptide 11
2.07	Cyp4a10	cytochrome P450, family 4, subfamily a, polypeptide 10
1.90	Cyp2e1	cytochrome P450, family 2, subfamily e, polypeptide 1
1.85	Cyp2c29	cytochrome P450, family 2, subfamily c, polypeptide 29
1.74	Ugt1a2	UDP glucuronosyltransferase 1 family, polypeptide A2 (3 copies)
1.57	Adh1	alcohol dehydrogenase 1 (class I)
		
		**Down-regulated**
		**Immune response and response to stress and inflammation**
		
3.60	Igk-V28	immunoglobulin kappa chain variable 28 (V28)
2.33	Serpina3n	serine (or cysteine) peptidase inhibitor, clade A, member 3N
1.93	Ngp	neutrophilic granule protein
1.90	Gp49a	glycoprotein 49 A /// leukocyte immunoglobulin-like receptor
1.68	Ifitm1	interferon induced transmembrane protein 1
1.63	Tirap	toll-interleukin 1 receptor (TIR) domain-containing adaptor protein
1.62	Gbp2	guanylate nucleotide binding protein 2
1.58	Fcgr2b	Fc receptor, IgG, low affinity IIb
1.52	Cd74	CD74 antigen (invariant polypeptide of MCH, class II antigen-associated)
1.50	Tnfaip6	
1.49	H2-Aa	tumor necrosis factor alpha induced protein 6
1.49	Aif1	histocompatibility 2, class II antigen A, alpha
1.46	Gbp4	allograft inflammatory factor 1
1.40	H2-D1	guanylate nucleotide binding protein 4
1.40	Hif3a ///	histocompatibility 2, D region locus 1
	LOC64109	hypoxia inducible factor 3, alpha subunit /// similar to hypoxia inducible factor 3, alpha subunit
		
		**Cell death and apoptosis**
		
2.15	Wwox	WW domain-containing oxidoreductase
1.65	Card10	caspase recruitment domain family, member 10
1.58	Hccs	holocytochrome c synthetase
1.55	Elmo1	engulfment and cell motility 1, ced-12 homolog (C. elegans)
1.39	Fadd	Fas (TNFRSF6)-associated via death domain
1.50	Hccs	holocytochrome c synthetase
1.39	Sphk1	sphingosine kinase 1

A number of genes identified in the arrays as significantly upregulated participate in the transport and metabolism of lipids and cholesterol. The up-regulation of LXR target genes involved in cholesterol efflux and transport such as ABCA1, Apolipoprotein A-II, Apolipoprotein C-III and several others was confirmed by RT-QPCR. A comparison between fold increase found in gene arrays and by RT-QPCR, as shown on Fig. 1A and 1B, demonstrates a correlation between the two assays. Three LXR target genes which were not upregulated in the arrays – ABCG1, apoE and SREBP1c, were also examined by RT-QPCR. As visible from Fig. 1A and 1B, ABCG1 was not up-regulated and apoE showed statistically significant but only a small (20%) increase. The only exception from the otherwise good overall correlation between the arrays and RT-QPCR data was the statistically significant (40%) increase in SREBP1c expression found by RT-QPCR, which was not surprising given the sensitivity of the method. The up-regulation of ABCA1 after extended administration of T0 was 1.6 fold as compared to more than 3.5-fold when T0 was applied for 24 hours (Fig. 1, compare A and D). The same difference, although to a smaller extent, was observed for other LXR target genes related to lipid metabolism, such as SREBP1c, apoE and ABCG1 (Fig. 1A and 1D), altogether suggesting that adaptive homeostatic mechanisms could be in place to modulate expression of ABCA1 and other LXR targets if T0 is applied for 25 days. The up-regulation of genes related to cholesterol efflux and transport also suggests that LXR ligands could affect the formation and the level of HDL-like lipoproteins in brain similarly to their effect in circulation.

The increased expression levels of several cytochrome P450 (Cyp450) enzymes and UDP glucoronosyl transferases, which are membrane microsomal enzymes, indicate an involvement of LXRs in the detoxification pathways and steroid (androgen and estrogen) metabolism. Some of these genes were previously identified by gene array assays as LXR targets in the liver and other tissues [28]. Changes in expression levels of transcription factors and genes involved in splicing and translation were also observed, suggesting that in APP23 at this age, LXRs may regulate transcription of genes via indirect mechanisms.

### T0 treatment affects the level of insoluble Aβ in APP23 mice

**Figure 2:**
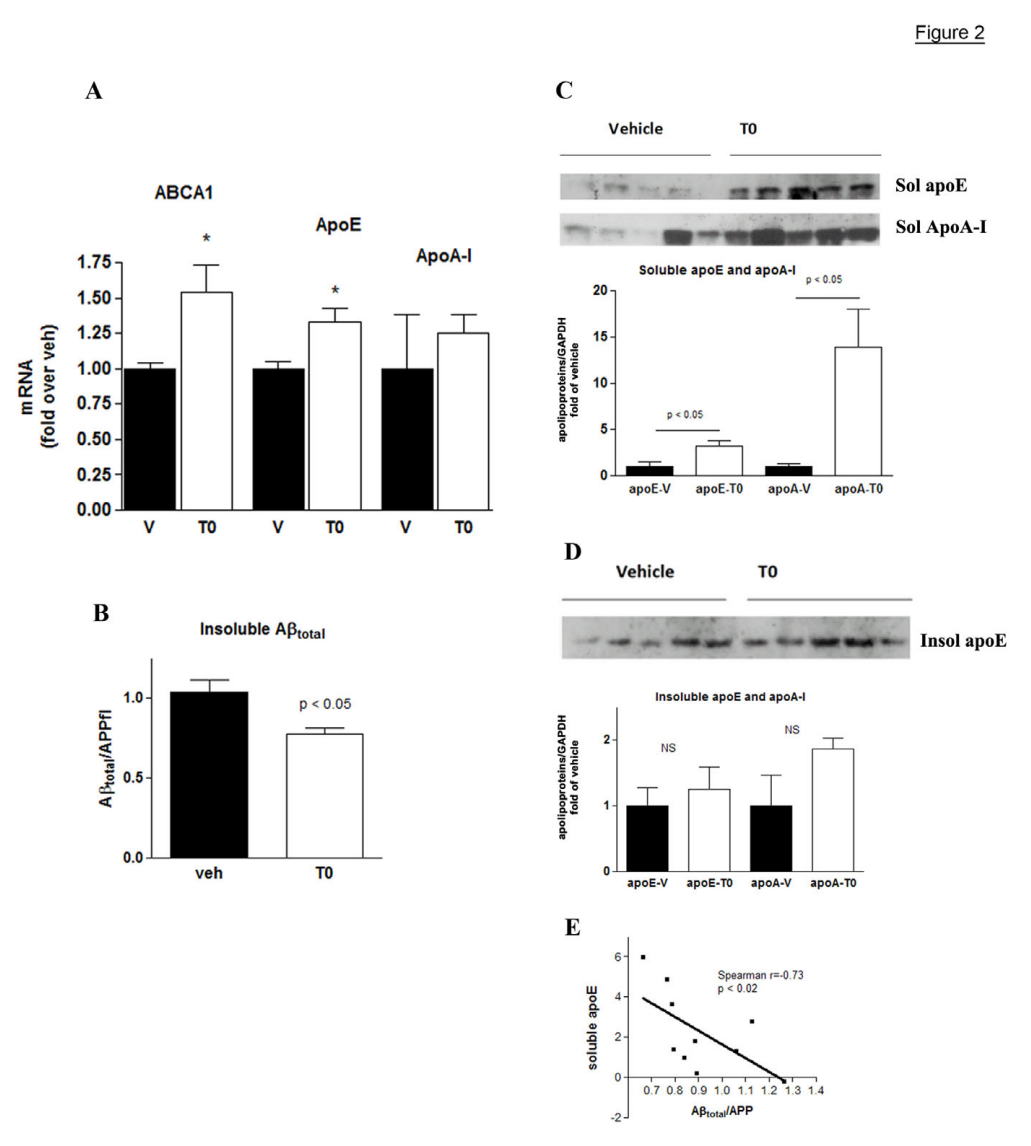
**Extended T0 treatment increases the levels of soluble apoE and apoA-I proteins and decreases insoluble Aβ in APP23 mice**. APP23 mice (n = 5) were treated by gastric gavage with T0 for 4 weeks at a dose of 20 mg/kg/day and age-matched control mice (n = 5) received vehicle. At the end of the treatment one hemisphere was used for total RNA isolation and the other was used for protein extraction. Soluble brain proteins were extracted with DEA followed by the extraction of the insoluble proteins from the pellet using formic acid. **A: **Expression level of ABCA1, apoE and apoA-I mRNA as determined by RT-QPCR. **B: **Insoluble Aβ_total _in aliquots from the insoluble brain fraction was determined by WB using by 6E10 antibody which recognizes both Aβ_40 _and Aβ_42_. The level of Aβ_total _was normalized to the level of APP full length (APPfl). **C: **Amounts of soluble apoE and apoA-I were determined by WB of formic acid extracted brain homogenates. The bands were quantified and the level of apoE normalized to the level of GAPDH. **D: **Insoluble apoE and apoA-I were determined by WB of formic acid extracted brain homogenate as in C. Values (A, B, C and D) are means ± SEM and represent fold of vehicle (two-tailed Student's *t *test). **E**. The level of insoluble Aβ_total _correlates negatively to the level of soluble apoE (Spearman Nonparametric correlation analysis).

Next we examined if the T0-mediated up-regulation of ABCA1 and apolipoproteins had an effect on the level of insoluble Aβ. T0 was administered to 6-month old APP23 mice (n = 5), 5 times per week for 4 weeks at a daily dose of 20 mg/kg body weight; the control group (n = 5) received vehicle. We decreased the dose of T0 in an attempt to minimize the liver steatosis which is a well known side effect of LXR ligands. At the end of the treatment, mRNA was isolated from the cortex and hippocampus of one hemisphere of each mouse and the activation of LXRs was confirmed by the increased level of ABCA1 mRNA in T0 versus vehicle treated mice (Fig. 2A). Cortices and hippocampi from the other hemisphere were homogenized and soluble and insoluble proteins were extracted, as before [14]. We were interested in the levels of apoE mRNA and protein because of its effect on Aβ aggregation and clearance [29,30] and we also examined the effect of LXR on apoA-I which was shown previously to affect Aβ aggregation in vitro [31]. ApoE and apoA-I in the soluble brain fraction (solApoE and solApo-A-I as determined by WB) of T0 treated mice were substantially increased: apoE more than 3 times and apoA-I more than 13-fold compared to their level in the same fraction of vehicle treated animals (Fig. 2C). In contrast, T0 treatment decreased significantly the level of total Aβ (comprising of Aβ_40 _and Aβ_42_) as measured by WB in the insoluble brain fraction using 6E10 antibody (Fig. 2B), while the level of soluble Aβ in the same set of samples was not changed (not shown). The increase in apoE protein level in T0 treated mice was accompanied by a small but statistically significant up-regulation of apoE mRNA (Fig. 1A). In contrast, whereas apoA-I protein level was increased by 13-fold, the increase of its mRNA was not statistically significant (1.6-fold, p < 0.2, Fig. 2A). It should be noted that we found similar results for ApoA-I on the microarrays: a 1.5-fold increase in apoA-I mRNA which was not statistically significant (p < 0.19, data not shown). In the insoluble fraction of brain homogenates of T0 treated mice there was no change in the level of apoE and the increase of apoA-I was not statistically significant (Fig. 2D). Finally, we found that there was a negative correlation between the levels of soluble apoE level and insoluble Aβ (Fig. 2E). This is in accordance with our studies using APP23/ABCA1^-/- ^mice, where the lack of ABCA1 caused an increase in amyloid load accompanied by a substantial decrease in soluble apoE level, with no change in the level of insoluble apoE [14]. We conclude that LXR activation increases the level of soluble apolipoproteins in the brain which correlates negatively to the level of insoluble Aβ.

### Cell type specific LXR regulation of apoE in brain

**Figure 3:**
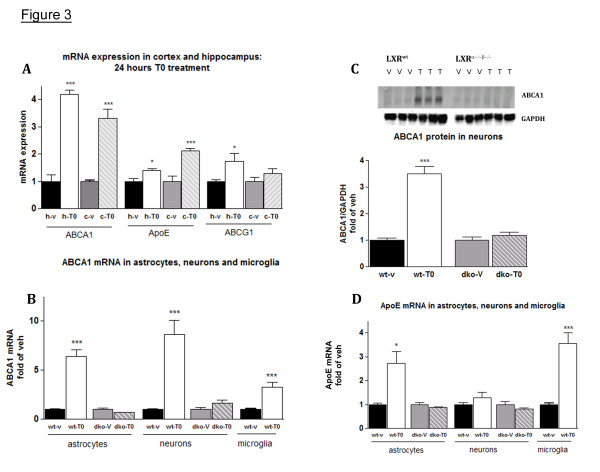
**Transcriptional regulation of apoE and ABCA1 expression by LXR in brain, glia and neurons**. **A: **7-month old APP23 mice were treated with 50 mg/kg T0 for 24 hours. Total RNA was extracted separately from cortices and hippocampi and mRNA expression of ABCA1, apoE and ABCG1 were determined by RT-QPCR; h-v, (black bars) – vehicle treatment, hippocampi; h-T0, (white bars) – T0 treatment, hippocampi, c-v (grey bars), vehicle treatment, cortex; c-T0 (hatched bars), T0 treatment, cortex. **B, C and D: **Primary neurons, astrocytes and microglia were established from wild type (wt) and LXRα^-/-^β^-/- ^double knockout mice (dko). Cells were treated with 10 μM T0 for 24 hours and mRNA expression of ABCA1 (**B**) and apoE (**D**) measured by RT-QPCR. Values are means ± SEM and represent fold of vehicle for the corresponding genotype. **C**: ABCA1 protein level in wild type (wt) and LXRα^-/-^β^-/- ^(dko) neurons was determined by WB. The level of ABCA1 was normalized to the level of GAPDH, and presented (means ± SEM) as fold of vehicle of the corresponding genotype in the graph bellow.

We interpret the increased level of brain apolipoproteins after T0 treatment as a combined effect of increased apoE and ABCA1 mRNA levels. The up-regulation of ABCA1 increases cholesterol efflux and consequently the lipidation of brain apoE and apoA-I, thus decreasing their catabolism. We were surprised, however, that the apoE mRNA up-regulation in the brain was rather small, even though statistically significant (Fig. 2A). One reason for this might be that LXR regulation is different in different brain areas. To test this hypothesis we treated 7 month old APP23 mice with T0 at a dose of 50 mg/kg body weight for 24 hours and examined the expression of several LXR target genes separately in the cortex and hippocampus (Fig. 3A). We specifically chose genes that have been previously implicated in the regulation of cholesterol efflux in brain – ABCA1, apoE and ABCG1. As shown in Fig. 3A, whereas T0 increased ABCA1 mRNA similarly in the cortex and hippocampus, apoE mRNA up-regulation was higher in the cortex than in the hippocampus. We did not find a corresponding change in apoE protein level in T0 treated mice as compared to vehicle treated ones (data not shown). Another reason for the smaller increase of apoE mRNA after T0 treatment might be that LXR ligands effectively up-regulate apoE mRNA expression only in a subset of brain cells. To examine this we treated primary astrocytic, microglial and neuronal cultures with T0 and examined ABCA1 and apoE mRNA expression. Fig. 3B shows that T0 (used at 10 μM concentration in this experiment) up-regulates ABCA1 expression in all three cell types in a similar way. To confirm that the observed effect is LXR dependent, we performed a series of experiments with wild type cells and cells obtained from LXRα^-/-^β^-/- ^double knockout (LXR^dko^) mice. We found that ABCA1 mRNA and protein levels were increased only in wild type cells (Fig. 3B and 3C, wt versus dko) confirming that the effect is LXR specific. In contrast, the LXR agonist up-regulated apoE mRNA expression only in astrocytes and microglia but not in neurons (Fig. 3D). Fig. 3D demonstrates that T0 increased apoE mRNA only in wild type astrocytes, confirming that apoE up-regulation was also LXR dependent. Therefore, whereas LXRs regulate ABCA1 in all brain cell types, their effect on apoE is cell-type specific which could explain the relatively small increase of apoE mRNA isolated from a total brain fraction.

### LXR agonists inhibit inflammatory response to LPS and fibrillar Aβ in microglia and astrocytes

**Figure 4:**
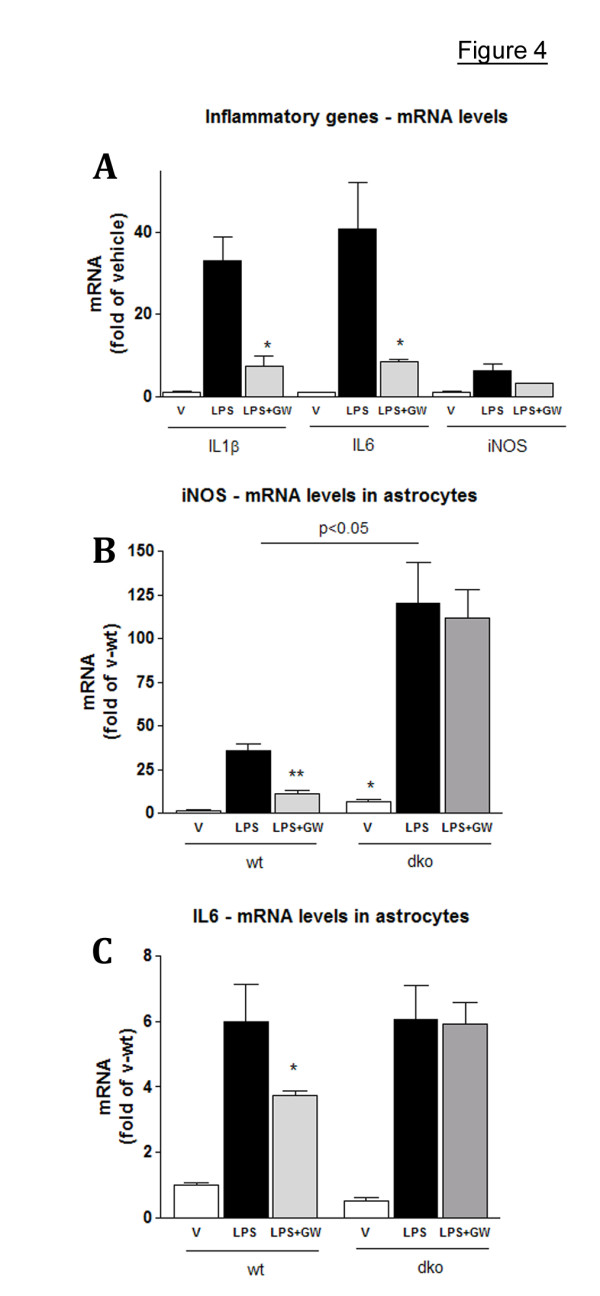
**LXR agonists down-regulate expression of pro-inflammatory genes in microglia and astrocytes**. Microglial cell line BV2 and astrocytes established from wild type (wt) and LXR^dko ^(dko) mice were pre-treated with LXR ligand GW or vehicle for 18 h prior to LPS treatment (50 ng/ml). Control cells received vehicle only (veh). For GW treated cells (LPS+GW) the ligand was co-applied with LPS. Cells were harvested 24 hours after LPS administration and the mRNA expression measured by RT-QPCR. **A: **BV-2 cells. Values are fold of vehicle. *, p < 0.05 LPS+GW treated compared to LPS only treated cells. **B and C: **WT and dko astrocytes were treated with LPS and GW as in A and mRNA expression of iNOS (**B**) and IL-6 (**C**) measured by RT-QPCR. Values (means ± SEM) are fold of wild type, vehicle treated cells of at least two independent experiments. Note that unlike in WT cells, GW does not decrease the expression of iNOS and IL-6 in dko cells. (LPS+GW in dko versus LPS+GW in WT, p < 0.01). For all experiments, LXR ligands were applied at 5 μM concentration. Statistics were performed by two-tailed Student's *t *test.

Our gene array data show that some genes related to immune response and inflammation, especially those related to the NF-κB cascade – such as Tirap and Serpina3n were down-regulated in APP23 mice after T0 treatment. In AD, CNS inflammation is a result of localized activation of microglia in areas surrounding the amyloid-β plaques and neurofibrillary tangles [32]. In addition, upon injury astrocytes assume an activated state associated with the release of inflammatory mediators [33]. It was shown that LXR ligands inhibit the expression of inflammatory mediators in peripheral peritoneal macrophages in response to lipopolysaccharide (LPS) stimulation [7]. Because the activation of microglia is believed to contribute to neurodegeneration by releasing proinflammatory and cytotoxic factors, including nitric oxide (NO) and IL-1β, we examined the anti-inflammatory effect of LXR ligands *in vitro *using microglia and astrocytes stimulated with LPS and Aβ. Fig. 4A demonstrates that GW-3965 (GW) (another widely used synthetic LXR agonist), used at 5 μM concentration down-regulated mRNA expression of the pro-inflammatory genes IL-1β, IL-6 and iNOS in the microglial cell line BV2. To examine if the decrease in mRNA expression is LXR dependent we used primary astrocytes derived from wild type and LXR^dko ^mice. As visible from Fig. 4B and 4C, GW inhibited LPS induced up-regulation of iNOS and IL-6 mRNA in wild type but not in dko astrocytes confirming that the down-regulation was LXR dependent. Moreover, there was a statistically significant difference in the expression level of iNOS and IL-6 between GW-treated wild type and dko cells (p < 0.01; compare LPS+GW in wt versus LPS+GW in dko in Fig. 4B and 4C).

**Figure 5:**
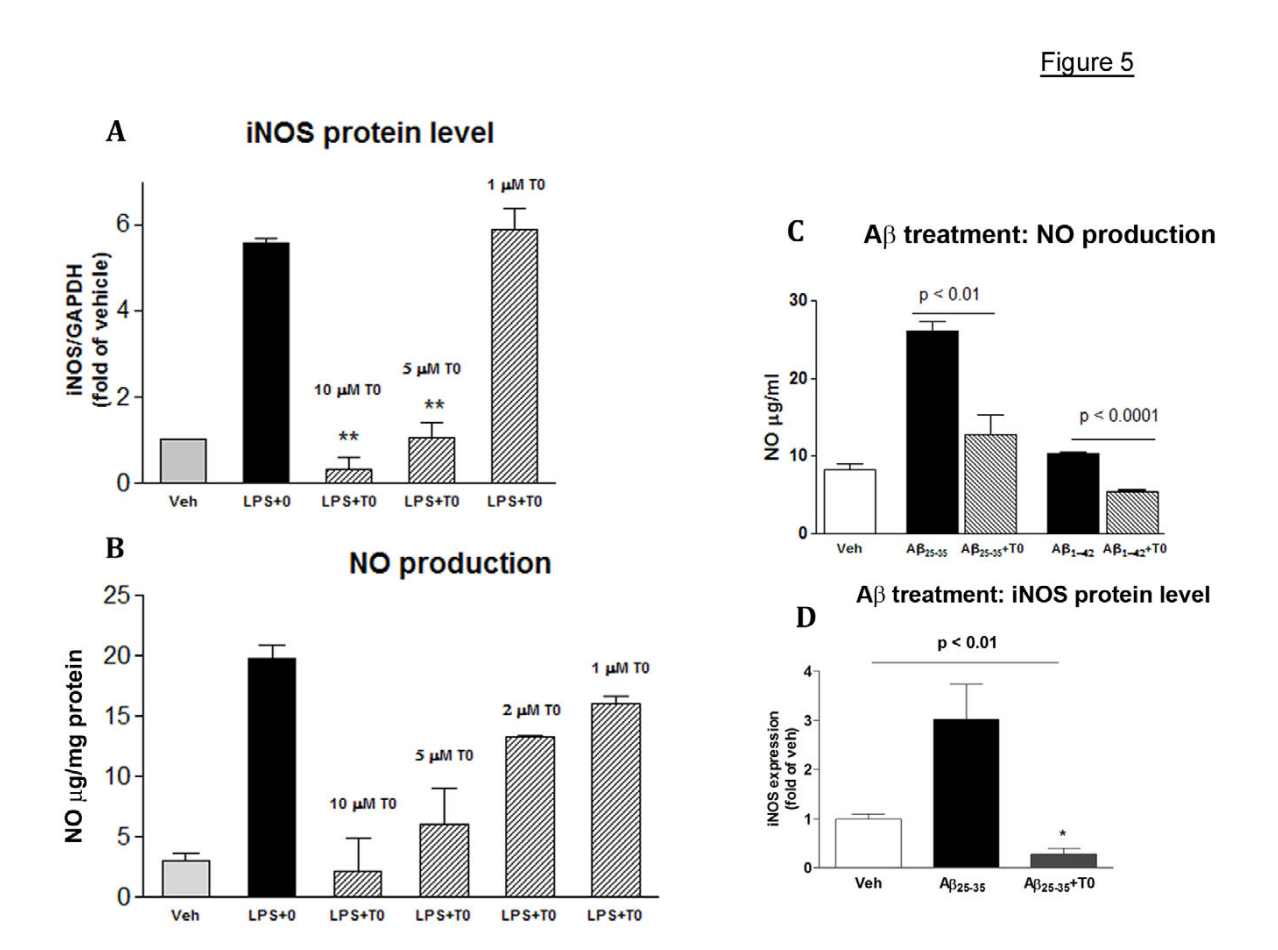
**T0 down-regulates LPS- and Aβ – induced iNOS synthesis and NO production in primary rat microglia**. Rat microglia was pre-treated with increasing concentration of T0 (LPS+T0) or vehicle (LPS only) prior to the addition of LPS (100 ng/ml) and the ligand was co-applied with LPS. Control cells received vehicle only (veh). Cells were harvested 24 hours after LPS administration and the expression of iNOS was examined by WB. **A and B: **Concentration dependent effect of T0 on LPS-induced iNOS protein and NO production in primary rat microglia. Microglia was pre-treated with increasing concentration of T0 (LPS+T0) or vehicle (LPS only) prior to the addition of LPS (100 ng/ml) and the ligand was co-applied with LPS. Control cells received vehicle only (veh). Cells were harvested 24 after LPS treatment and the expression of iNOS was examined by WB **(A)**. NO in the conditioned media was measured by Griess reagent **(B)**. **C and D: **T0 (10 μM) inhibits NO production (**C**) and iNOS protein (**D**) induced by fibrillar Aβ_25–35 _or Aβ_42 _peptides applied for 24 hours to microglia. Note that T0 decreases iNOS even bellow its basal level if applied with Aβ (in **D**, veh versus Aβ_25–35_+T0, p < 0.01). Values are means ± SEM; two-tailed Student's *t *test; *, p < 0.05.

Next, we examined if LXR ligands decrease the secretion of proinflammatory cytokines in primary rat microglial cells treated with LPS or fibrillar Aβ_25–35 _and Aβ_42 _peptides. First, we examined the effect of T0 on nitric oxide (NO) production and iNOS protein level which are normally increased in response to LPS. As shown on Fig. 5A and 5B, T0 applied 18 hr prior to and then co-applied with LPS down-regulated the expression of iNOS and NO production in rat microglia in a concentration-dependent manner. Similarly, T0 (used at 10 μM concentration) inhibited the expression of iNOS in Aβ treated cells and production of NO in the conditioned media (Fig. 5C and 5D). Noticeably, T0 decreases iNOS protein even bellow its basal level (p < 0.01, vehicle versus Aβ_25–35_+T0 in Fig. 5D).

**Figure 6:**
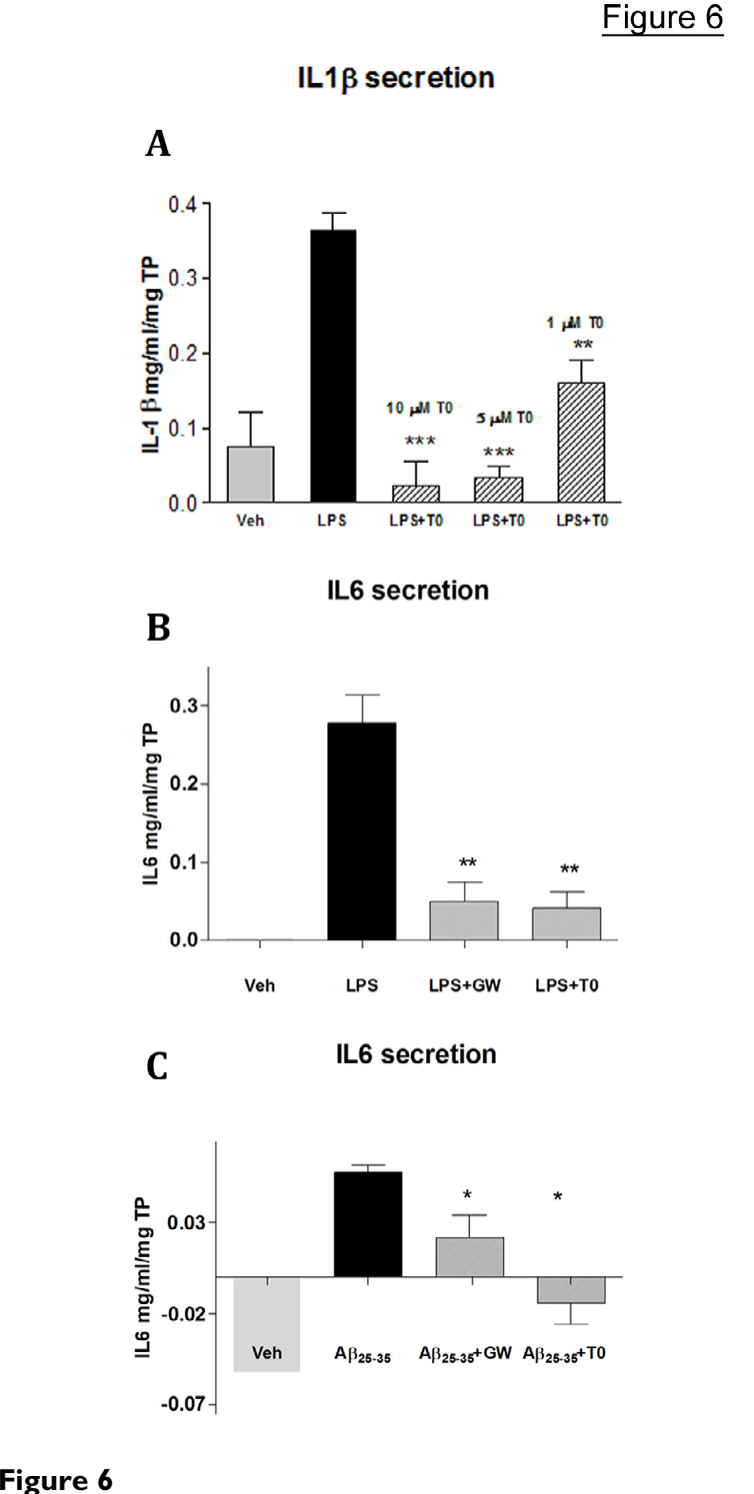
**LXR ligands decrease the secretion of pro-inflammatory cytokines in primary rat microglial cells treated with LPS or fibrillar Aβ_25–35_**. **A: **Rat microglia were treated with increasing concentrations of T0 and LPS as in Fig. 5 and IL1-β secretion measured by ELISA. **B **and **C**: Rat microglia were treated with 5 μM T0 and GW and then LPS (B) or Aβ_25–35 _**(C) **as in Fig. 5; IL-6 secretion was measured by ELISA. The values (means ± SEM) indicate the concentration of the cytokines in mg/ml and are normalized to the total cellular protein (TP). LXR treatments are compared to LPS or Aβ_25–35 _alone by two-tailed Student's *t *test.*, p < 0.05, **, p < 0.01, and ***, p < 0.001.

Because the results of our experiments shown on Fig. 4 demonstrate downregulation of the inflammatory cytokines IL-1β and IL-6 mRNA, we also examined the effect of LXR ligands on their secretion induced by LPS or fibrillar Aβ. As visible from Fig. 6A LPS increased the secretion of IL-1β more than 5 fold and T0 suppressed this effect in a dose-dependent manner. The basal secretion of IL-6 in these cells was undetectable but LPS and aggregated Aβ induced it to a measurable level with Aβ having a smaller effect. Again, the application of either of the two LXR ligands – T0 or GW (used at 5 μM), decreased the production of IL-6 from these cells.

### T0 treatment decreases the expression of pro-inflammatory genes in APP23 mice

**Figure 7:**
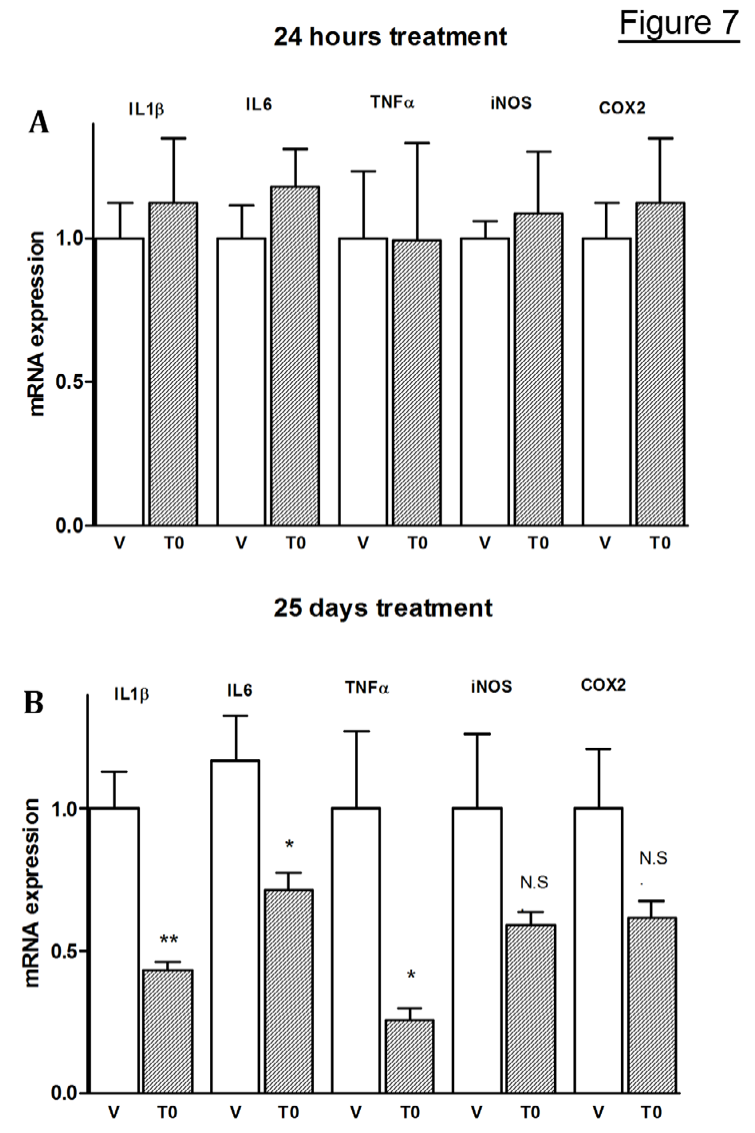
**T0 decreases the expression of pro-inflammatory genes in APP23 mice**. 7 month old APP23 mice (n = 5) were treated by gastric gavage with T0 at a dose of 50 mg/kg/day for 24 hours (A) or 4 weeks (B), 6 times a week and age-matched control mice (n = 5) received vehicle. At the end of the treatment total RNA was isolated from cortices and hippocampi and mRNA expression of indicated genes determined by RT-QPCR. Values are means ± SEM and represent fold of change compared to vehicle treatment (two-tailed Student's *t *test).

To examine further the effect of LXR ligands on brain inflammation we used APP23 mice at age of 7 moths. At this age there is a substantial increase of aggregated Aβ in their brain paralleled by inflammatory reactions [34]. We applied T0 (50 mg/kg/day) to mice randomized in two separate groups (n = 5 for both): the mice in the first group were treated with T0 for 24 hours and those in the second for 25 days every day. At the end, the expression of several pro-inflammatory genes was measured by RT-QPCR. Fig. 7A shows that short-time T0 treatment of APP23 mice did not affect the expression of the examined pro-inflammatory genes. In contrast, extended T0 treatment of APP23 mice decreased significantly mRNA expression of IL-1β, IL-6 and TNFα. The expression of iNOS and COX2 showed a trend towards a decrease although the difference was not significant (Fig. 7B). We conclude that LXR ligands decrease the expression of certain pro-inflammatory genes induced by amyloid deposits *in vivo *only if administered for a longer period of time. At present, it is unclear if LXR ligands directly suppress the transcription of these genes or the effect is indirect.

## Discussion

Epidemiological, clinical and experimental studies have suggested a link between cholesterol metabolism and AD pathogenesis [35-41]. Despite the therapeutic potential of this link, the mechanisms by which cholesterol metabolism influences AD pathogenesis are still unclear. LXR target genes control the removal of excess cholesterol through efflux, catabolism or decreased absorption, lipid metabolism and lipoprotein remodelling [4]. Because of their regulatory effect on different aspects of lipid metabolism it has been speculated that LXR ligands might impact metabolic disease and atherosclerosis particularly. We have reported that a short treatment, 6 days, of young APP23 transgenic mice with the synthetic LXR ligand T0 significantly decreased the level of both Aβ_40 _and Aβ_42 _[21]. Using another APP transgenic model (Tg2576) or WT mice and applying T0 for up to 14 days, two other groups found a decrease in Aβ_42 _but not in Aβ_40 _levels [19,42]. At the age of treatment, the mice used in the second study were pre-deposing, i.e. lacking insoluble Aβ, and therefore the observed effect was a result of a decreased level of soluble Aβ peptides.

In the present study we wanted to determine which genes are affected on the expression level by extended T0 treatment and if the administration of T0 to APP23 mice decreases amyloid deposition at the age when plaque formation begins. Our results show that prolonged administration of T0 at 50 mg/kg/day in APP23 mice up-regulated a number of LXR target genes related to lipid metabolism. A previous study exploring transcriptional profiling of T0 treated non-APP transgenic mice identified some of these genes as differentially up-regulated in the brain of LXR^wt ^but not in LXR^dko ^mice [28]. In terms of AD phenotype, we observed that T0 mediated activation of LXR increased protein level of soluble apoE and apoA-I in the brain which correlated negatively to the level of insoluble Aβ. This is in agreement with our previous studies in APP/ABCA1^-/- ^mice where the elevation of amyloid load was accompanied by considerable decrease of soluble brain apoE [14]. At the same time the magnitude of the increase of apoE protein did not correspond to the rather small increase of apoE mRNA as identified in gene arrays, and by RT-QPCR. One reason for this is that apoE is selectively up-regulated in different brain cell types – in glia but not in neurons. Similarly, in peripheral cells apoE is transcriptionally regulated by LXR in macrophages and adipocytes but not in liver cells [43]. We speculate that the increased protein level of apoE following T0 treatment is a combination of: (1) LXR mediated transcriptional up-regulation of apoE and (2) transcriptional up-regulation of ABCA1 in glia that leads to an increased cholesterol efflux. The net effect is an increased level of properly lipidated brain apoE.

In brain the lipidation of apoA-I, which comes primarily from the circulation, depends on ABCA1, as it does in the periphery and similarly to the mechanism for apoE lipidation, which has been extensively discussed in relation to Aβ deposition and clearance [2,12,14,16]. There are no published data that LXRα/β exert a regulatory role on apoA-I expression at the transcriptional level in mouse or human cells and cell lines, although, such a regulatory mechanism has been demonstrated in chick embryo hondrocytes [44]. Nevertheless, T0 administered to APP23 mice causes an increase in apoA-I transcript (albeit not statistically significant) and protein. The results from our study allow an explanation, based on the so-called antagonizing effect of T0 on NF-κB, which results in down-regulation of NF-κB responsive genes [7]. It has been shown that cytokines exert a strong inhibitory effect on apoA-I expression at the mRNA and protein levels in both human and mouse primary cells, established cell lines, and *in vivo *[45,46]. Our Affymetrix gene array and RT-QPCR assays clearly demonstrate that a prolonged application of T0 to APP23 mice ultimately causes a decrease in TNFα and IL-6 expression levels. While ABCA1 upregulation and ABCA1-facilitated cholesterol efflux are the primary mechanisms for apoA-I lipidation and its increased stability, we consider the sustained inhibition of cytokines as one additional mechanism for elevated expression of apoA-I and thus an increased amount of soluble apoA-I in brain.

Properly lipidated and therefore stable apoE and apoA-I, are an indispensable part of functional HDL-like lipoprotein complexes in brain. The legitimacy of this hypothesis is confirmed by the studies demonstrating that LXR agonist treatment of mice increases plasma levels of HDL, HDL particle size and apoA-I protein [47] – effects attributed to the increased ABCA1 expression and cholesterol efflux. In brain the increased expression of ABCA1 could lead to: (1) an increase in the amount of Aβ bound to lipid-rich apoE, which may have a significant role in maintaining the level of soluble Aβ and preventing it from aggregation, and (2) an increased delivery of Aβ to astrocytes for degradation, or Aβ clearance through the blood brain barrier. Support for this hypothesis is provided by a recent study [27], which revealed an increased plaque load in APP transgenic mice with global deletion of LXRα or LXRβ, and further reinforced the idea that LXRs and their responsive genes are important determinants in AD pathogenesis [2,14,20].

Our results from gene array assays demonstrated that a number of genes related to immune response and inflammation were down-regulated in the brain of APP23 mice. This group of genes was not identified previously [28] as differentially affected by T0 in LXR^wt ^or LXR^dko ^probably because the animals in that study were not APP expressing transgenic mice. In the course of AD, CNS inflammation is an invariant finding and is considered a result of a localized activation of microglia by fibrillar or oligomeric Aβ. AD brain with active chronic neurodegeneration is primed to elicit faster and more pronounced proinflammatory responses to central and systemic inflammatory challenges. The up-regulation of inflammatory genes identified by microarray in incipient AD supports such a notion [48]. Moreover, a recent study demonstrated that with aging, without any neurodegenerative pathology, there was an up-regulation in immune/inflammatory pathways [49]. Different protein markers including MHC class I and class II proteins, IgG Fc-gamma receptors, pro-inflammatory cytokines and tumor necrosis factor alpha (TNF-α) were found upregulated in AD and some of those were down-regulated by LXR ligand in our gene array assays. Similar data have been recently published by Zelcer et al [27]. Using primary glia, the authors demonstrated up-regulation of proinflammatory genes in response to fibrillar Aβ and a strong inhibitory effect of GW. In their study expression profiling of GW treated BV2 cells revealed up- or down-regulation of genes clustered in two groups primarily related to lipid metabolism and transport, while LPS treatment of the cells strongly induced a battery of inflammatory genes, which were potently repressed if GW was added to the culture.

In our study, Serpina3n is a gene that was down-regulated (more than 2-fold, Fig. 1C) after extended T0 treatment of APP23 mice and may have an impact on amyloid deposition. Serpina3n is a murine homolog of human α-Antichymotrypsin and is one of 13 closely related inhibitors within the murine serpina3 cluster [50]. In brain, α-Antichymotrypsin is a component of the acute inflammatory response. Human α-Antichymotrypsin has been identified in amyloid plaques and was shown to promote Aβ aggregation as well as tau phosphorylation [51-53]. In this study we also demonstrate that LXR ligands have anti-inflammatory effect in primary microglia and astrocytes activated by LPS or fibrillar Aβ. Most importantly we found that T0 inhibits the expression of pro-inflammatory cytokines in the brain of APP23 mice after extended but not short-term (24 hours) application.

## Conclusion

Many observations substantiate the hypothesis that the presence of activated microglia in vulnerable areas of AD brains accelerates the undergoing pathological processes presumably triggered by soluble and fibrillar Aβ aggregates and deposits. It is becoming increasingly evident that LXR controlled genes are involved in many steps of AD pathogenesis, which makes LXRs promising therapeutic targets in AD. An increased understanding of the LXR controlled regulation of Aβ aggregation and clearance systems will lead to the development of more specific and powerful agonists targeting LXR for the treatment of AD.

## Methods

### Chemicals

T0901317 was obtained from Cayman Chemicals (Ann Arbor, Michigan). The following were purchased from Sigma (Saint Louis, MO): LXR ligand GW-3965 (GW), lipopolysaccharide (LPS) from *Salmonella typhimurium*, delipidated calf serum, leupeptin, aprotinin, and 4-(2-aminoethyl)benzenesulfonyl fluoride hydrochloride (AEBSF). Aβ_1–42 _and Aβ_25–35 _peptides were purchased from Bachem (King of Prussia, PA). Tissue culture flasks and plates were from Corning (Corning, NY) and Falcon (Lincoln, NJ). In stock solutions T0 and GW were dissolved in ethanol at a 1,000-fold final concentration. The source of all cell culture reagents, if unspecified, was Invitrogen (Carlsbad, CA).

### Aβ peptides and aggregation conditions

Aβ_1–42 _and Aβ_25–35 _peptides were dissolved in H_2_O to a concentration of 250 mM. Fibrillar Aβ were generated by incubating 250 μM of different Aβ peptides for 6 days with shaking at 37°C, as previously described [31] and additionally diluted in culture medium to the desired final concentration before addition to the cell culture. Fibril formation was monitored by Thioflavine-T fluorescence and Congo red measurements [31].

### Antibodies

Rabbit polyclonal anti-ABCA1 antibody was purchased from Novus (Littleton, CO). The 6E10 monoclonal antibody (Signet, Dedham, MA) recognizes the first 17 amino acids of the Aβ peptide. This antibody was used for Western blotting (WB) of total Aβ and to detect full-length human APP and sAPPα. Rabbit C8 polyclonal antibody [21] was used to detect CTF resulting from α – or β-secretase cleavages. Rabbit 869 antibody [21] was used to detect sAPPβ by WB. Murine-specific polyclonal apoE antibody was from Santa Cruz (Santa Cruz, CA), polyclonal murine-specific anti-apoA-I antibody was from Rockland Immunochemicals, Inc. (Gilbertsville, PA), anti-inducible nitric oxide synthase (iNOS) monoclonal antibody was from BD Biosciences (San Jose, CA) and Glyceraldehyde-3-phosphate dehydrogenase monoclonal antibody was purchased from Chemicon International (Temecula, CA). The following antibodies were used for ELISA: (1) Aβ – anti-Aβ40 (G2–10 mAb) and anti-Aβ_42 _(G2–13 mAb) monoclonal antibodies conjugated to horseradish peroxidase were from Genetics Company (Schlieren, Switzerland); (2) capture and detection antibodies for IL-1β were from Biosource International Inc., (Camarillo, CA). ELISA kit (Duoset) for IL-6 was from R&D Systems, Inc (Minneapolis, MN). Secondary antibodies conjugated to horseradish peroxidase were from Jackson ImmunoResearch (West Grove, PA).

### Transgenic mice

The study fully conformed to the guidelines outlined in the Guide for the Care and Use of Laboratory Animals from the U.S. Department of Health and Human Services and was approved by the University of Pittsburgh Institutional Animal Care and Use Committee. We used APP23 transgenic mice (C57BL/6 background) expressing human familial AD mutant APP751 with Swedish double mutation at positions 670/671 (APPK670N, M671L). The expression of human APPsw is driven by the murine Thy-1 promoter and is restricted to neurons [54].

### In vivo treatments

5–7 month old APP23 mice were treated by gastric gavage with T0 dissolved in propylene glycol/Tween 80 (4/1) as in [21] for: (1) 25 days with 50 mg/kg/day (n = 5); (2) 5 times per week for 4 weeks at a dose of 20 mg/kg/day (n = 5); (3) 50 mg/kg for 24 hours (n = 5). Control mice (n = 5 for each treatment group) received vehicle. It is well known that T0 treatment increases the level of triglycerides in the liver causing steatosis. We measured liver weight (T0 versus veh) after completion of each T0 treatment as a criterion for steatosis. 24 hours treatment didn't increase the liver weight. 50 mg/kg/day of T0 applied for 25 days increased liver weight in treated animals by 84% and 20 mg/kg/day for 30 days by 40% (all data, relative to vehicle treated animals.)

### Animal tissue processing

Mice were asphyxiated with CO2 and perfused transcardially with 30 ml PBS (0.1 M, pH 7.4). Brains were rapidly removed, the olfactory bulb and cerebellum deleted, and hemibrains were snap-frozen on dry ice. Where indicated, cortices and hippocampi were dissected and processed separately.

### RNA isolation and Affymetrix gene array assay

Total RNA from homogenized cortices and hippocampi was isolated using TRIzol reagent according to the manufacturers protocol (Invitrogen) with ethanol precipitation and a second clean-up using Qiagen RNeasy mini kit. The integrity of the RNA samples was assessed qualitatively on Agilent 2100 Bioanalyzer to assure high quality (ratios for 28S to 18S bands close to 2:1) of total RNA. One Cycle cDNA synthesis Kit (Affymetrix) was used for first and second strand cDNA synthesis. Cleanup, and synthesis of biotin-labelled cRNA for target labelling assays was accomplished by GeneChip IVT labelling kit (Affymetrix). Following cleanup and quantitation, cRNA was fragmented for target hybridization and probe arrays were hybridized for 16 hours. Washing and staining were automatically performed on Fluidics Station 450 and the arrays were scanned on GeneChip^® ^Scanner 3000. All subsequent statistical analyses have been performed on original CEL files containing raw probe intensity data for each array using Array Assist software (Stratagene, CA). Functional annotation clustering analysis was performed using DAVID Bioinformatics Resources 2007 (National Institute of Allergy and Infectious Diseases, NIH).

### Real Time Quantitative PCR

Total RNA from brain tissue was isolated and purified as described for gene array assays. For TaqMan^® ^based RT-QPCR first-strand cDNA was synthesized using Sprint™ PowerScript™ PrePrimed Single Shots (Clontech). All TaqMan^® ^gene expression assays were performed using ready to use probe and primer sets for the corresponding genes on ABI 7500 Real-Time PCR System (Applied Biosystems). Amplification plots were analyzed by Comparative ΔCt method with mouse GAPDH or cyclophilin as housekeeping genes [55]. Primer sequences are available upon request.

### Protein extraction, Western blotting and ELISA

To extract proteins, brains (150 mg/ml buffer) were homogenized in Tris/sucrose buffer (250 mM sucrose, 20 mM Tris base, 1 mM EDTA, 1 mM EGTA, pH 7.4) using a glass vessel and piston-type Teflon pestle as previously described [2,56]. Protein extracts were prepared: (1) RIPA – 1:1 dilution of the initial homogenate with RIPA buffer (10 mM Tris-HCl, pH 7.3, 1 mM MgCl_2_, and 0.25% SDS, 1% Triton X-100) in the presence of protease inhibitors (10 μg/ml leupeptin, 10 μg/ml aprotinin and 10 μg/ml AEBSF), sonicated and used for Western blotting (WB) for ABCA1 and full length APP as before (cit); (2) soluble and insoluble brain proteins (Aβ and apolipoproteins) were extracted essentially as described previously [2,56]. Briefly, soluble proteins were extracted using cold 0.4% diethylamine in 100 mM NaCl, spun at 135,000 × *g *for 1 h at 4°C, and neutralized by adding 0.5 M Tris-HCl, pH 6.8. The pellet was further extracted using 70% cold formic acid, sonicated for 1 min, spun as above, and neutralized (1 M Tris base, 0.5 M Na_2_HPO_4_, and 0.05% NaN_3_).

WB for insoluble Aβ was performed using 10 μl of formic acid extracted Aβ. The solvent (formic acid) was evaporated and the pellet resuspended in 2 × NUPAGE loading buffer. The proteins were resolved on 4–12% Bis Tris NUPAGE gels and transferred on the nitrocellulose membranes. Membranes were boiled for 5 min in PBS and Aβ_total _was detected with 1:1000 dilution of 6E10 antibody. Soluble Ab was measured in RIPA extracted fraction by WB using 6E10. Soluble Ab was also measured in diethylamine extracted protein fraction by ELISA performed using 6E10 as the capture antibody and anti-Aβ_40 _(G2–10 mAb) and anti-Aβ42 (G2–13 mAb) monoclonal antibodies conjugated to horseradish peroxidase as detection antibodies. The amount of Aβ was normalized either to the total protein or to the expression of APPfl as measured by WB. To detect soluble apoA-I and apoE we used diethylamine extracted protein fraction. WB for insoluble proteins was performed as before [2], using 10 μl of formic acid extracted insoluble fraction as described above for Aβ. 10% Tris-Glycine gels were used to resolve soluble and insoluble apoA-I and apoE.

### Primary cell culture

All primary cells were obtained from dissociated cortices and hippocampi. Primary neurons were derived from 17–18-day-old wild type or LXR^dko ^mouse embryos as described previously [20]. Briefly, cells were derived from cortices and hippocampi (dissociated with 1× trypsin-EDTA for 10 min at room temperature) and cultivated in Neurobasal medium supplemented with B27, GlutaMax II (Invitrogen) and antibiotics. Neurons were plated at high density (2 × 10^5^/ml, 1 ml/well) on poly-D-lysine (100 μg/ml) coated 12 well Costar plates and used at Day in Vitro 5. Primary mouse (C57BL/6J) and rat microglial cells were derived from newborn mouse or rat pups respectively using the same protocol and essentially as before. Astrocytes were derived from newborn pups of wild type or LXRα^-/-^β^-/- ^and used for few passages. Microglia and astrocytes were cultivated in DMEM with 10% FBS. Protein expression level of ABCA1 and apoE was measured by WB in RIPA extracted cell lysates using the respective antibodies.

### Inflammatory response in BV2 cells, microglia and astrocytes

The experiments were performed essentially as before [7,57]. Briefly, cells (in DMEM with 5% delipidated serum) were pre-treated with LXR ligands 18 hours prior LPS or Aβ treatment and then the ligands were co-applied with LPS or Aβ. Cells were treated 24 hours with LPS (50 ng/ml) or Aβ (50 μM). The protein expression level of iNOS was measured in the cell lysate by WB. Nitric oxide production was measured in the conditioned media using Griess assay [7]. The secretion of IL-1β and IL-6 was examined in the conditioned media using mouse ELISA kits from Biosource and R&D respectively as suggested by the manufacturer.

### Statistical analysis

Results are reported as mean ± SEM. Statistical significance was determined by two-tailed Student's *t *test. We used Spearman correlations to determine the unadjusted relationships between continuous variables (GraphPad Prism software, Windows version 4.0, San Diego, CA).

## Competing interests

The author(s) declare that they have no competing interests.

## Authors' contributions

RK, AB and IL carried out all experiments. ZW contributed significantly to the work with LXR knockout mice, primary cultures and RT-QPCR. DM and MS contributed conceptually to the design of some experiments and helped to draft the manuscript. IL and RK are corresponding authors, contributing intellectually with the design, conception of the project, analysis and interpretation of the data, and manuscript writing. All authors read and approved the final manuscript.
